# Comprehensive analysis and classification of retrocondylar ulnar groove morphology using CT imaging in an average population of adults

**DOI:** 10.1007/s00276-024-03297-x

**Published:** 2024-02-08

**Authors:** Axel Przyklenk, Michael Hackl, Tim Leschinger, Nadine Ott, Andreas Harbrecht, Lars Peter Müller, Kilian Wegmann

**Affiliations:** Department of Orthopedic and Trauma Surgery, Faculty of Medicine and University Hospital Cologne, University of Cologne, Kerpener Straße 62, 50937 Cologne, Germany

**Keywords:** Retrocondylar ulnar groove, Computed tomography, Elbow, Radiologic anatomy

## Abstract

**Purpose:**

Anatomical variations of the concave shaped retrocondylar ulnar groove (RUG) can contribute to ulnar nerve instability. However, there are currently limited available standardized data describing the anatomy of the RUG based on radiologic imaging, such as computed tomography (CT). This study aims to provide a comprehensive description and classification of RUG anatomy based on RUG angle measurements.

**Methods:**

400 CT scans of the elbows of adults showing no signs of osseous damage were evaluated. RUG angles were measured in four anatomically defined axial planes that spanned from the proximal to the distal end of the RUG. Furthermore, distance measurements at the medial epicondyle were conducted. A classification system for the RUG is proposed based on the acquired RUG angles, aiming to categorize the individual angles according to the 25th and 75th percentiles.

**Results:**

RUG angles were significantly larger in males compared to females (*p* < 0.001) accompanied by larger distances including the off-set and height of the medial epicondyle (*p* < 0.001). RUG angles decreased from proximal to distal locations (*p* < 0.05).

**Conclusion:**

This study revealed that men exhibited larger RUG angles compared to women, indicating a less-concave shape of the RUG in men. Introducing an objective RUG classification system can improve our understanding of anatomical variations and potentially find application in diagnostics and preoperative planning.

## Introduction

The pathogenesis of ulnar nerve instability, including dislocation or subluxation of the ulnar nerve, remains a topic of debate. It has been observed that during elbow flexion at approximately 90°, the unstable ulnar nerve can dislocate medially from its stabilizing retrocondylar ulnar groove (RUG) [17]. Consequently, a shallow RUG has been suggested as a significant anatomical factor that promotes ulnar nerve instability [1, 18, 19]. However, there is a lack of standardized data on the native anatomy and geometric shape of the RUG in the elbow of healthy adults, particularly using modern imaging techniques such as computed tomography (CT).

Previous studies, though limited in number, have reported that the prevalence of ulnar nerve instability with associated dislocation or subluxation ranges from 20 to 30% among asymptomatic healthy adults [8, 9, 14]. However, prolonged ulnar nerve dislocation leads to frictional forces acting on the nerve, increasing the likelihood of symptomatic ulnar neuropathy or neuritis [6, 8, 20]. Several surgical approaches, including anterior transposition of the ulnar nerve, medial epicondylectomy, or ulnar groove plasty, have been described to address these patients [18]. However, the underlying pathology and risk factors for developing ulnar nerve instability have not been fully understood or clearly defined, particularly regarding anatomical factors contributing to this condition. To facilitate individualized treatment for ulnar nerve instability and improve the clinical outcomes of potential surgical interventions, it is crucial to establish normal values describing the native shape of the RUG at various anatomically defined locations.

Addressing this important need, the purpose of this study is to describe the shape of the RUG in the elbows of adult men and women at different anatomically defined locations, based on CT scans. Furthermore, utilizing these findings, the study aims to propose a classification system for the RUG that offers comprehensive information on its shape.

## Methods

Measurements were performed on multi-planar reconstructed CT scans of the elbow of adults in a retrospective manner. The measurement protocol was developed by experienced trauma surgeons and conducted by a radiology-trained member of the surgery department. The interpreter had no information on the patient’s medical history. The study was approved by our institution’s Review Board (22–1236-retro) and did not require informed consent due to its retrospective nature.

### CT scans

We randomly selected CT scans that met the following criteria: epiphyseal closure, no direct fracture signs, arthrosis or implants at the distal humerus, and CT slice thickness ≤ 1.0 mm. These CT scans were performed between 2011 and 2016 for clinical indications other than our study, such as suspected osseous damage following elbow trauma (e.g., luxation, fall, and polytrauma). Only CT scans that showed no distal humerus damage were used.

All CT scans were performed using the iCT 256 scanner (Philipps Healthcare, Cleveland, OH, USA) and the SOMATOM Force scanner (Siemens Healthcare, Forcheim, Germany).

CTs were applied using 120 kVP accompanied by a gantry rotation time of 0.5 s and a single collimation width of 0.625 mm for all scans as well as varying slice thickness of 0.85 mm ± 0.08 mm (mean ± standard deviation), 164.5 mAs ± 48.4 mAs, a field of view of 216.0 mm ± 65.8 mm, pixel size of 0.30 mm ± 0.08 mm, a matrix size of 711.1 (± 99.1) × 711.1 (± 99.1) pixels, and a spiral pitch factor of 0.38 ± 0.20.

### Study design

400 CT scans were measured according to a standardized measurement protocol. Intra-rater-reliability was calculated via re-measurement of 100 CT scans that were selected randomly. Re-measurement was conducted in an interval of 21 days. Upon collecting CT data, the RUG classification was computed.

### Measurement protocol

All measurements were conducted using the IMPAX EE software (AGFA Health Care, Mortsel, Belgium).

#### CT standard planes

The axial, coronal, and sagittal planes are adjusted according to the following standard. The coronal plane parallels the longitudinal humerus shaft axis (LHSA) in the sagittal view and is adjusted parallel to the joint line (connection between most volar location on the capitulum and medial trochlea lip in axial view) on the deepest location of the olecranon fossa in the axial view. The sagittal plane is adjusted parallel to the LHSA of the distal humerus shaft in the coronal view. The axial plane bisects the trochlea in the sagittal view as the sagittal plane is adjusted intersecting the trochlea groove.

#### Longitudinal humerus shaft axis

To define the longitudinal humerus shaft axis (LHSA), the ‘centerline by four points’ tool was used in both sagittal and coronal view through the IMPAX EE software (AGFA Health Care, Mortsel, Belgium). The four points were adjusted based on a standardized method [15].

In the sagittal view, a line was drawn between the most proximal location on the outer cortical surface of the distal humerus and the most volar location of the joint surface of the trochlea which was also used as a surrogate of depicted distal humerus length. In the coronal view, the line was drawn between the most proximal location of the lateral outer cortical surface of the distal humerus and the most lateral location on the lateral epicondyle. Points 1–4 were positioned on the outer cortical surface. Point 1 is adjusted on the most proximal location as described above. Point 2 is located perpendicular to the middle of the connection line. For the adjustment of point 3 and 4, a line is drawn connecting point 1 and 2. Point 3 and 4 are located perpendicular to this line, while point 3 is adjusted opposite to point 1 and point 4 opposite to point 2. The minimum and maximum values of the distal humerus length were standardized to 50 mm and 100 mm, respectively, to standardize the humerus shaft axis adjustment and enable the use of elbow CT images. CT images with a distal humerus length < 50 mm were excluded, and those > 100 mm had point 1 shifted distally to produce a humerus shaft length of 100 mm [15].

### Distance measurements in the coronal plane

**Fig. 1:**
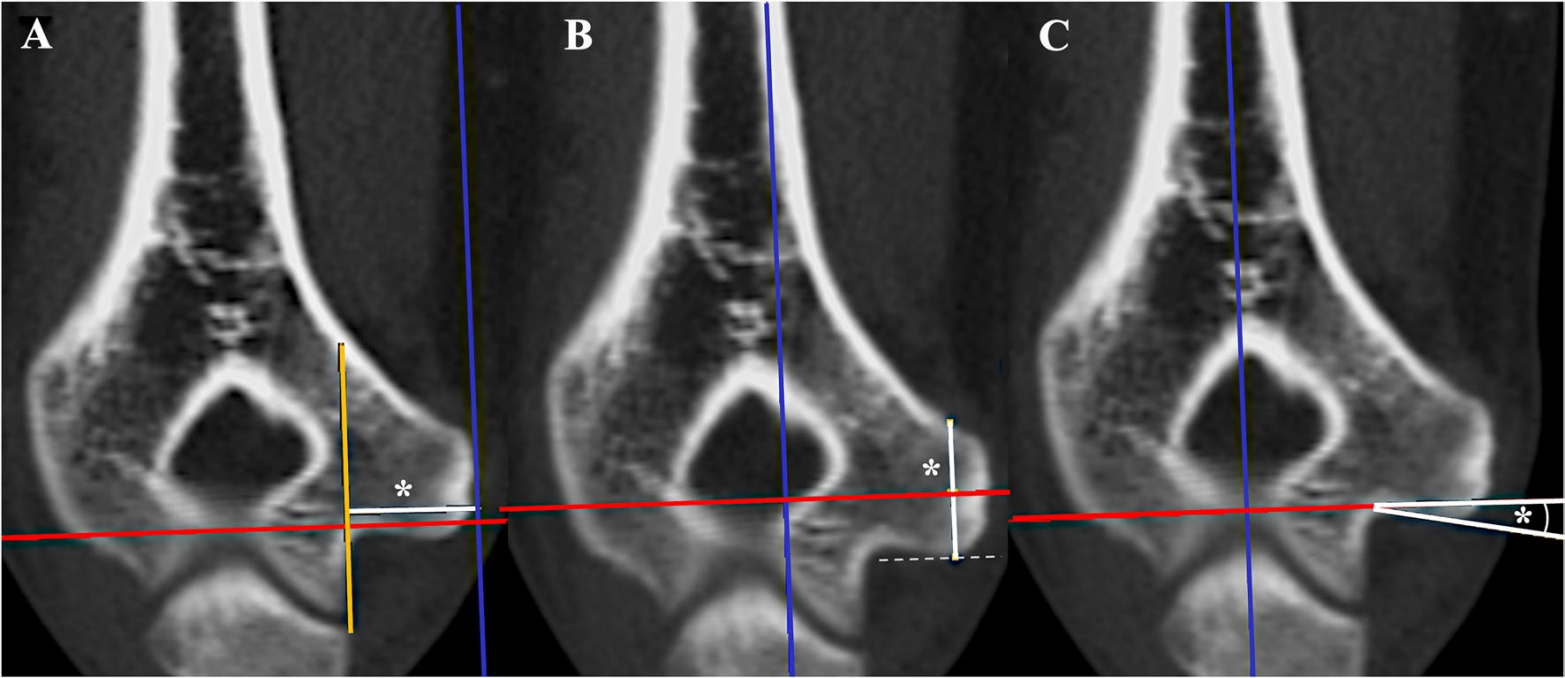
Measurements in the coronal plane. Off- set of the medial epicondyle (ME) (**a**), height of the ME (**b**), and the coronal angulation of the inferior ME (**c**) are outlined. *: Point of measurement; blue line: sagittal plane; red line: axial plane. Orange line in (**a**) is drawn parallel to the sagittal plane and represents the medial boundary of the trochlea, while the sagittal plane is adjusted on the most medial point of the ME. The dotted line in **b** indicates the most distal point on the ME. All pictures are taken in the same coronal plane of the same CT scan

Standard planes may not be rotated. The coronal plane may not be moved. Trans-epicondylar distance (TED) was measured as the maximal distance between the outer surface of the radial and ulnar humeral epicondyles [7]. The off-set of the medial epicondyle (ME) was defined as the distance between the most medial location on the ME and the medial boundary of the trochlea. The distance was measured parallel to the axial plane and orthogonal to the sagittal plane (Fig. 1a). The height of the ME was measured parallel to the sagittal plane and orthogonal to the axial plane starting from the most distal location on the ME (Fig. 1b). The coronal angulation of the inferior ME was measured in the coronal plane between the axial plane and a line connecting the most proximal location in the coronal ulnar groove and the most distal location on the ME (Fig. 1c).

### RUG angles

**Fig. 2:**
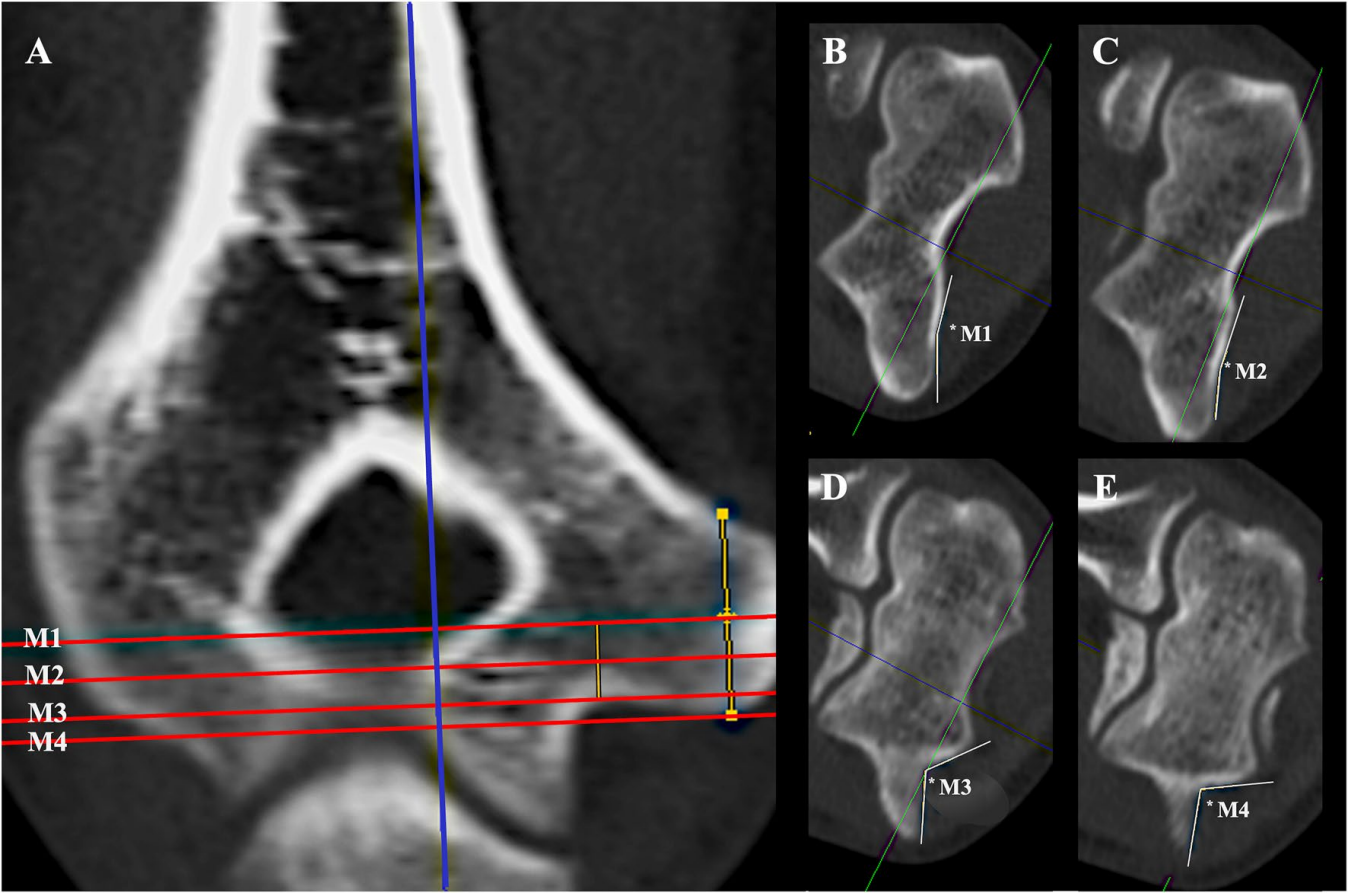
Retrocondylar ulnar groove angle measurements. Measurement locations are adjusted in the coronal view (**a**) by locating the middle of the medial epicondyle (M1), the most proximal point in the ulnar groove (M3), the middle between M1 and M3 (M2), and the most distal point on the medial epicondyle (M4). The corresponding axial view for M1 (**b**), M2, (**c**), M3 (**d**), and M4 (**e**) are shown including the location of angle measurement (*). Blue line: sagittal plane; red line: axial plane; green line: coronal plane. Orange lines: distance measurements serving the location of M1 and M2

The RUG angles are measured in the axial view. While the coronal and sagittal planes may not be moved, the axial plane is adjusted in the coronal view according to the defined measurement locations. Measurement locations (Fig. 2a) are the middle of the height of the ME (M1), the most proximal location in the ulnar groove (M3), middle of the distance between M1 and M3 (M2), and the most distal location on the ME.

Angle measurements are conducted between two lines connecting the angular point which is the deepest (most volar) point of the RUG in the axial view with the lateral and medial transitions from a concave to a convex shape (Fig. 2b–e).

### RUG classification

**Fig. 3:**
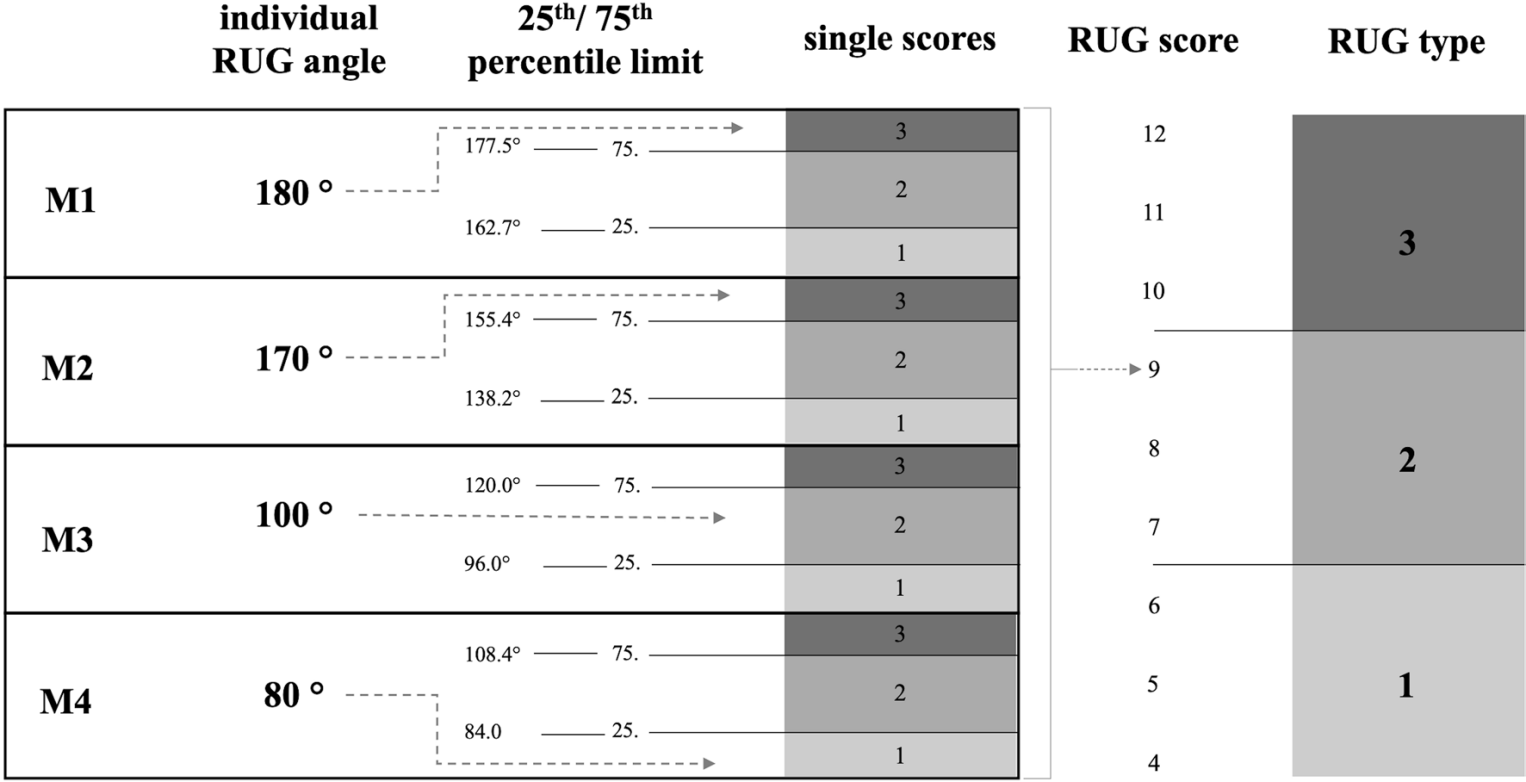
Classification of the retrocondylar groove (RUG) based on a single case. The arrows indicate the transformation of individual RUG angles at the locations M1, M2, M3, and M4 into single scores based on the 25th and 75th percentile limit. The percentile limits are derived from this study (Table 1). The RUG score is a sum score of all four single scores that is used to describe the RUG type. In the example presented, the combination of the individual RUG angles corresponds to an RUG classified as type 2

We aim to describe three RUG types that are built through a computed RUG score that aids to describe the morphologic anatomy of the RUG including four standardized axial planes (M1–M4). Higher scores are associated with higher RUG angles thus reduced concavity of the RUG. For better understanding, an example of an individual RUG classification is depicted in Fig. 3.

**Table 1:** Sex-unspecific descriptive data of retrocondylar ulnar groove angles

	Angle at M1 [deg °]	Angle at M2 [deg °]	Angle at M3 [deg °]	Angle at M4 [deg °]
Mean	168.1	144.8	108.9	96.8
Standard deviation	13.6	16.7	18.5	20.2
Minimum	63.7	57.2	41.8	37.1
25th percentile	162.7	138.2	96.0	84.0
Median	171.5	147.2	110.3	95.2
75th percentile	177.5	155.4	120.0	108.4
Maximum	196.1	177.3	188.1	164.0

Angles for all measurement locations (M1, M2, M3, and M4) are listed in angle degrees (deg °)

#### RUG score

The RUG score (Fig. 3) is determined by summing individual scores calculated for each axial RUG angle measurement location (M1, M2, M3, M4). To compute the single scores, angle values smaller than the 25th percentile limit are assigned a score of ‘1’, those between the 25th and 75th percentile are assigned a score of ‘2’, and values greater than the 75th percentile are assigned a score of ‘3’. Consequently, the RUG score ranges from 4 to 12, representing the summation of the single scores obtained from M1, M2, M3, and M4.

#### RUG types

We have defined three distinct RUG types (Fig. 3) by categorizing the RUG score into three value ranges. RUG type 1 encompasses scores ranging from 4 to 6, type 2 includes scores from 7 to 9, and type 3 comprises scores from 10 to 12.

### Statistical analysis

SPSS software (version 12.2; IBM, Chicago, Illinois) was used for all analyses. Normal distribution was tested applying the Kolmogorov–Smirnov test; normal distribution was assumed when *p* > 0.05. Unpaired t tests or Mann–Whitney *U *tests were applied to test for group differences (male vs. female) regarding the variables TED, off-set of the ME, height of the ME and coronal angulation of the inferior ME. A two-way analysis of variance (ANOVA) (factor 1: measurement location [M1; M2; M3; M4]; factor 2: sex [male; female]) was used to test for sex-differences between RUG angles at each measurement location and to detect angle differences between measurement locations within the groups (male; female); Bonferroni post hoc test was applied to measure for significances (*p* < 0.05). The eta coefficient was used for correlations between sex and other variables; significance was tested via one-way ANOVA (factor: sex [male; female] (*p* < 0.05). The Spearman correlation coefficient was computed for correlations between age, TED, off-set of the ME, height of the ME, coronal angulation of the inferior ME, and the RUG angles; correlations were significant when *p* < 0.05. Intra-rater variability was calculated via Spearman correlation between first and second measurement of 100 randomly selected CTs.

## Results

### Patient characteristics

400 CT scans including 232 men and 168 women were measured. The female collective (47.4 ± 18.3 years) was older compared to males (42.0 ± 14.5 years) (*p* < 0.05).

### Measurements in the coronal plane

**Fig. 4:**
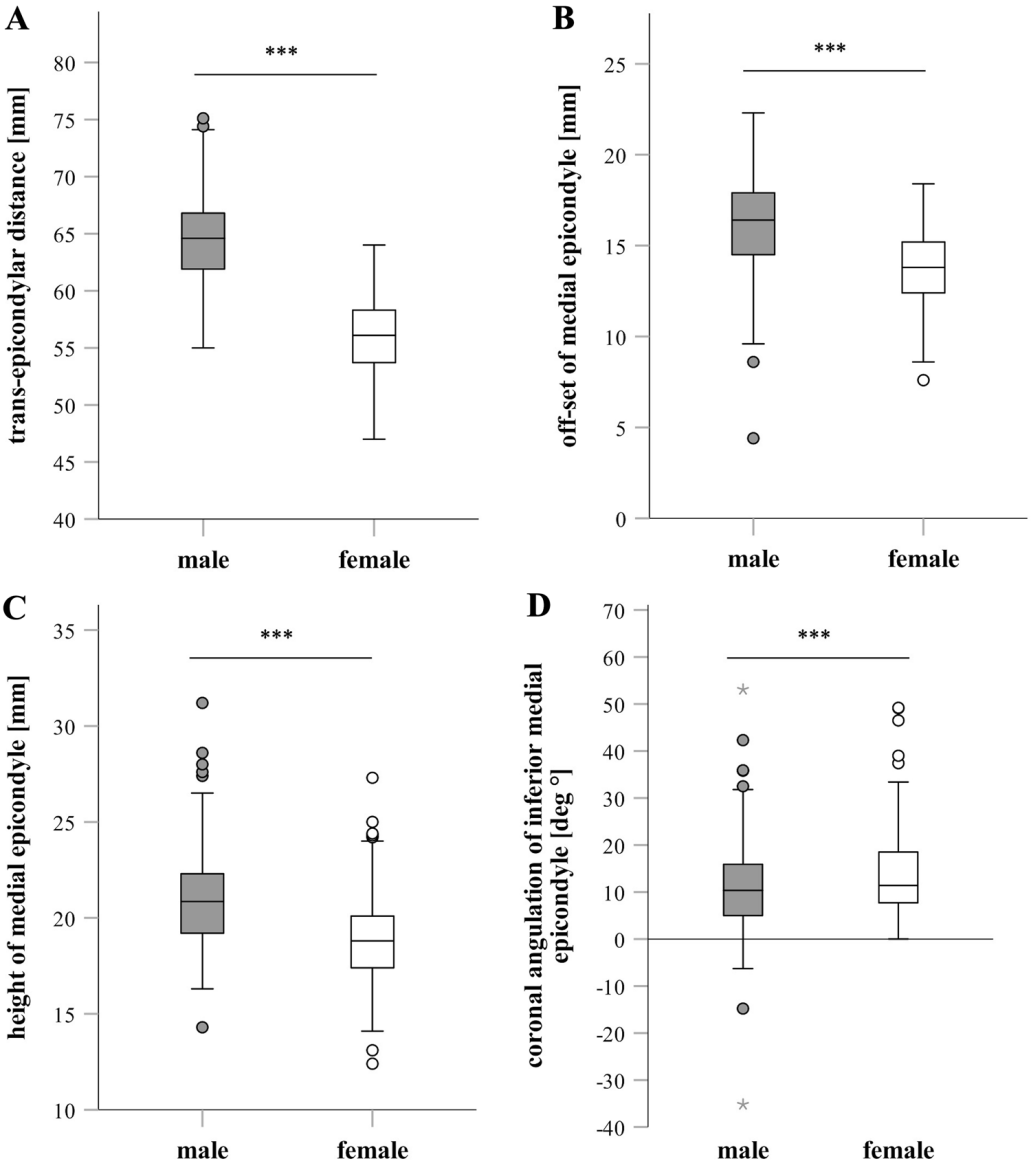
Measurements in the coronal plane. Trans-epicondylar distance (**a**), off-set (**b**), and height of the medial epicondyle (**c**) as well as the coronal angulation of the inferior medial epicondyle (**d**) are depicted as Box–Whisker plots (minimum, 25th percentile, median, 75th percentile, maximum, and outliers) comparing males and females. *** Significant differences between males and females (*p* < 0.001)

While the coronal angulation of the inferior ME was higher in female compared to male subjects (*p* < 0.001), TED, off-set, and the height of the ME were significantly higher in male compared to female subjects (*p* < 0.001) (Fig. 4).

### RUG angles

**Fig. 5:**
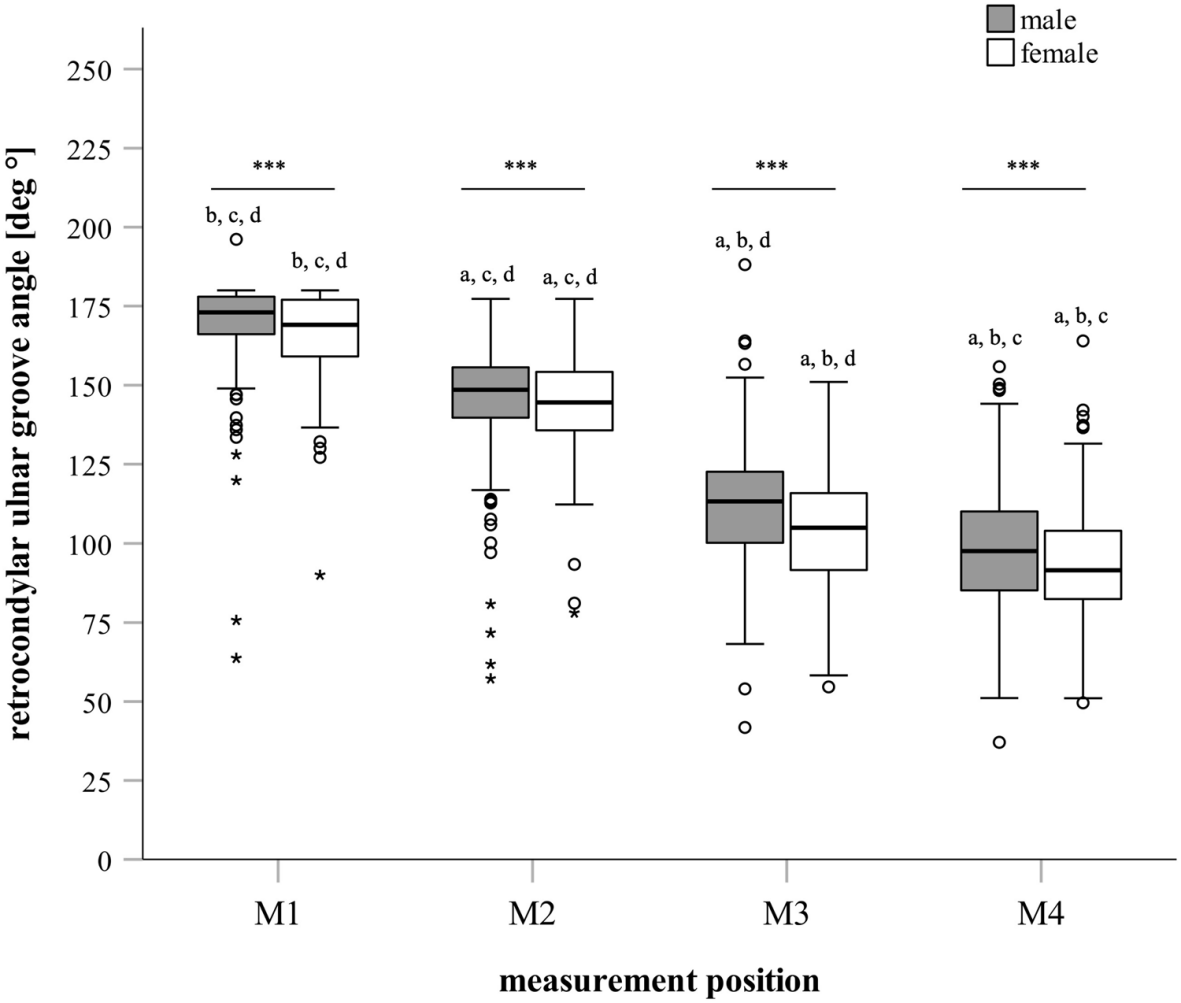
Retrocondylar ulnar groove angles. Angles are depicted as Box–Whisker plots (minimum, 25th percentile, median, 75th percentile, maximum, and outliers) for all measurement locations (M1, M2, M3, M4) grouped by sex (male, female). ***: significant difference between males and females at measurement location (*p* < 0.001); *a*: significant differences vs. M1 in same group; *b*: significant differences vs. M2 in same group; *c*: significant differences vs. M3 in same group; *d*: significant differences vs. M4 in same group (*p* < 0.001)

For descriptive data of RUG angles, see Tables 1 and 2. Angles at all measurement locations differed significantly from each other (*p* < 0.001) with decreasing angles from M1 to M4 (Fig. 5). Angles were significantly higher in men compared to women at M3 and M4 (*p* < 0.001). Although angle values at M1 and M2 were also higher in men vs. women, significance was not reached (M1: *p* = 0.065; M2: *p* = 0.226).

### Correlations

Sex correlated significantly with the TED (eta coefficient = 0.76; *p* < 0.001), off-set of the ME (eta coefficient = 0.44; *p* < 0.001), height of the ME (eta coefficient = 0.40; *p* < 0.001), coronal angulation of the inferior ME (eta coefficient = 0.13; *p* = 0.009), and RUG angles at M1 (eta coefficient = 0.12; *p* = 0.019), M3 (eta coefficient = 0.20; *p* < 0.001), and M4 (eta coefficient = 0.14; *p* < 0.005).

Moderate Spearman coefficients [2] (*r* ≥ 0.5) were found for correlations between TED and height of ME (*r* =  + 0.48, *p* < 0.001), TED and off-set of ME (*r* =  + 0.68, *p* < 0.001), off-set of ME and outer UG angle (*r* =  + 0.64, *p* < 0.001), coronal angulation of inferior ME and retrocondylar angle at M4 (*r* =  − 0.50, *p* < 0.001), retrocondylar angles at M1 and M2 (*r* =  + 0.47, *p* < 0.001), M2 and M3 (*r* =  + 0.48, *p* < 0.001), and M3 and M4 (*r* =  + 0.65, *p* < 0.001). Age did not correlate significantly (*p* > 0.05) with any of the investigated variables.

### Intra-rater reliability

Intra-rater reliability was ‘excellent’ [10] for all measured variables with Spearman correlation coefficient *r* > 0.9 (*p* < 0.001).

### RUG classification

**Fig. 6:**
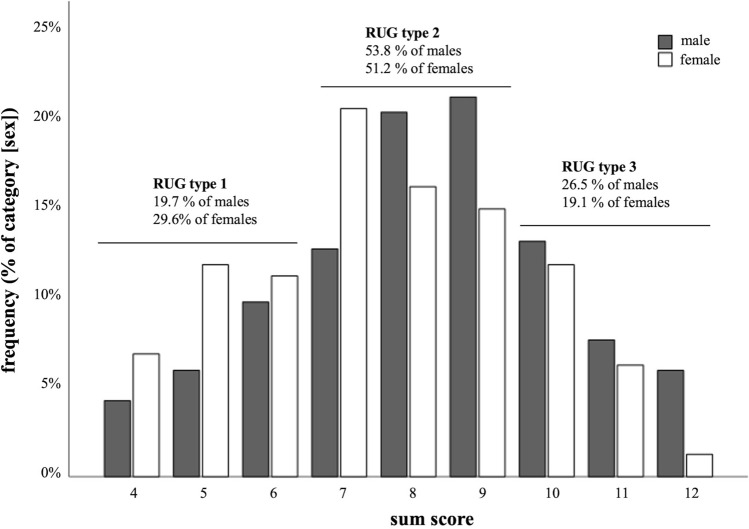
Retrocondylar ulnar groove sum scores and resulting types. Frequencies are depicted as percentage of the respective category (male, female)

Using the 25th and 75th percentile values for sex-unspecific RUG angles (Table 1), our findings indicate the most common RUG (sum) scores to be 7 for women and 9 for men (Fig. 6). RUG type 2 is observed in approximately 50% of individuals from both sexes. Notably, RUG type 3 is the second most prevalent among men, while RUG type 1 represents the second highest proportion among women (Fig. 6).

## Discussion

A shallow retrocondylar ulnar groove (RUG) is believed to contribute to ulnar nerve instability, along with other anatomical factors such as adjacent tissues [1, 19]. As the shallowness of the RUG is defined by its depth which can be influenced by differences in bone dimensions, we chose to describe the geometry of the RUG using angles instead of depth. However, the existing literature lacks normal values for the axial angulation of the RUG and an objective description. Therefore, our objective was to establish normal axial values of the RUG of a large population using CT imaging. This allowed us to compare the RUG between men and women and develop an objective classification system for RUG morphology to aid in identifying RUG abnormalities.

Overall, our findings demonstrate that axial RUG angles gradually decrease from proximal to distal planes, indicating an increased concavity of the RUG closer to the inferior border of the medial epicondyle (ME). Interestingly, our correlational analysis revealed a significant relationship between sex and RUG angles, as well as ME angulation. Thus, we compared RUG angles and ME angulation between women and men, which showed that men had greater RUG angles and a smaller (coronal) ME angulation, indicating a reduction of concavity of the RUG compared to women. Unfortunately, there are no studies describing the exact geometry of the RUG, making it difficult to directly compare our results with the existing literature. However, a shallow RUG is commonly believed to contribute to ulnar nerve instability [4, 19]. Although we did not measure RUG depth, it could be hypothesized that a reduction of RUG concavity might result in a decrease of the osseous stabilization of the ulnar nerve in men. However, other studies have not found differences in the frequency of ulnar nerve sub- or dislocation between men and women [4, 19]. Despite the tendency of women to seek medical attention more frequently than men, which may potentially underestimate the prevalence of ulnar nerve instability in men [5, 16], it is important to acknowledge the presence of other anatomical factors that contribute to ulnar nerve stability. These factors could act as counterweights, effectively balancing the less-concave RUG observed in men and thereby aiding in the prevention of ulnar nerve instability [9]. Thus, one explanation for the absence of increased rates of ulnar nerve luxation in men could be that the travel distance of the ulnar nerve from the RUG to anterior dislocate over the ME is simply longer in men due to a larger dimensioned medial epicondyle. This hypothesis finds support in our detection of a greater off-set of the medial epicondyle in men compared to women which might be accompanied by a larger depth of the RUG in men (not measured).

By obtaining angle measurements at multiple axial CT slices, we achieve a comprehensive evaluation of the entire osseous anatomy of the RUG, ensuring a more precise assessment of RUG morphology and any potential variations along its length. The importance of a comprehensive CT imaging allowing to investigate multiple thin slices of osseous structures was also highlighted by previous investigations on the bony structure of the cubital tunnel and the medial epicondyle anatomy [3, 11, 12]. For example, Lee et al. (2020) investigated the angle of the bony floor of the cubital tunnel spanned between the medial boarder of the trochlea and the inferior border of the medial epicondyle at the maximal cubital tunnel depth, while they rotated the measurement plane around the cubital tunnel center. Thereby, they highlighted the importance of multiple slice measurements as they delivered ranging values with varying planes of measurement in individuals of a population of healthy adults and in patient with cubital tunnel syndrome [11].

Understanding the significant contribution of the RUG to ulnar nerve stability at the medial epicondyle, we are the first to propose an anatomical classification of RUG morphology based on CT images. While Mirza et al. (2021) have considered reduced depth of the RUG in the classification of ulnar nerve instability as a disadvantageous factor, the description of the RUG has lacked standardized classification criteria [13]. Instead of radiologic depth or angle measurements, Mirza et al. (2021) included the depth of the RUG in their classification through intraoperative naked-eye evaluation [13]. While such approaches may be suitable in the intraoperative setting, with experienced surgeons being able to distinguish between shallow, normal, or deep RUGs, it introduces subjectivity. Therefore, our CT-based classification of the RUG establishes an objective measurement system that can supplement such classifications.

However, while we aim to describe the anatomy of the RUG through an overall RUG type, future studies may find it superior to correlate the presence of ulnar nerve instability with individual RUG scores or single angles. These RUG scores can potentially identify specific RUG morphologies favoring ulnar nerve instability that might be missed when applying our overall RUG type in clinical correlational studies—for example, higher RUG scores at specific locations that might represent a higher disadvantage for ulnar nerve stability compared to higher scores at other RUG locations.

The complexity of our angle assessment, necessary for producing reliable data, cannot be ignored. While an experienced observer can perform one measurement in all four axial planes, along with the coronal measurement of the medial epicondyle (ME) off-set and ME angulation, in under 10 min, extensive training is required to achieve acceptable measurement times for possible clinical implementation. Nevertheless, as diagnostic and preoperative planning continues to advance, the identification of abnormal RUG angles could offer valuable insights into managing potential ulnar nerve pathologies. This study primarily aimed to provide a detailed description of the bony anatomy of the RUG based on CT imaging. In combination with studies on patients with ulnar nerve pathologies focusing on additional anatomical aspects such as soft-tissue variations affecting the stabilization of the ulnar nerve, our data could potentially contribute to the development of diagnostic tools that could be capable of identifying the causes of specific pathologies or symptoms and, accordingly, personalizing therapy options.

## Limitations

Addressing the limitations of this study, it is important to note that while measuring angles at multiple axial CT slices offers significant advantages in evaluating RUG morphology, integrating this technique with clinical assessments is crucial. Thereby, the influence of the osseous morphology of the RUG together with soft-tissue variations on ulnar nerve instability and dislocation as well as the development of ulnar neuropathy should be studied including anthropometric and ethnic data in future approaches. To ensure the reproducibility of RUG angle measurements, we have extensively standardized our measurement methodology. However, this standardization has increased the time required for individual measurements, with each measurement taking initially approximately 15 min and necessitating thorough practice to reach acceptable measurement times. Additionally, we have not assessed inter-observer reliability as we observed high inter-observer agreement during the development of our methodology. However, future studies should evaluate inter-observer reliability among experts at different levels. In our study, we were unable to utilize the true longitudinal humerus shaft axis (LHSA) due to the limited field of view in elbow CT scans, which is applied to minimize X-ray exposure. Although we did not find a significant correlation between the depicted humerus shaft length and the measured angles during the development of our methodology, it is recommended that future studies examine the relationship between our study’s LHSA and the true LHSA.

## Conclusion

In conclusion, our study utilizes CT imaging to establish normal axial angle values of the RUG, revealing a less-concave RUG in men compared to women. Additionally, the introduction of an objective classification system for RUG morphology enhances our understanding of anatomical variations of the RUG and has the potential to be employed in diagnostic and advanced preoperative planning.

## Data Availability

The data that support the findings of this study are available from the corresponding author, Axel Przyklenk, upon reasonable request.
